# Repurposing Rosiglitazone Induces Apoptosis Accompanied by Impaired Antioxidant Defense in Cholangiocarcinoma Cells: Findings from Proteomic and Functional Analyses

**DOI:** 10.3390/ph19010044

**Published:** 2025-12-24

**Authors:** Benjaporn Buranrat, Prakasit Sa-Ngiamwibool, Auemduan Prawan, Sarinya Kongpetch, Piman Pocasap, Laddawan Senggunprai

**Affiliations:** 1 Biomedical Sciences Research Unit, Faculty of Medicine, Mahasarakham University, Maha Sarakham 44000, Thailand; benjaporn.b@msu.ac.th; 2 Department of Pathology, Faculty of Medicine, Khon Kaen University, Khon Kaen 40002, Thailand; prakasa@kku.ac.th; 3 Department of Pharmacology, Faculty of Medicine, Khon Kaen University, Khon Kaen 40002, Thailand; peuamd@kku.ac.th (A.P.); sarinyako@kku.ac.th (S.K.); pimapo@kku.ac.th (P.P.); 4 Cholangiocarcinoma Research Institute, Khon Kaen University, Khon Kaen 40002, Thailand

**Keywords:** rosiglitazone, PPARγ, cholangiocarcinoma, apoptosis, redox homeostasis, proteomic analysis

## Abstract

**Objectives**: The repurposing of existing drugs as anticancer agents has attracted attention in cancer drug discovery. This study aimed to examine the anticancer efficacy of rosiglitazone (RSG) against cholangiocarcinoma (CCA) and its underlying mechanisms. **Methods**: The effect of RSG on the viability of KKU-100 CCA cells was examined. The possible molecular targets were identified using proteomic analysis and verified by a series of cell-based assays. Furthermore, the expression of PPARγ protein in CCA tissues was also assessed. **Results**: RSG exhibited a cytotoxic effect against KKU-100 cells. Proteomic analysis demonstrated a significant different expression protein pattern of the 100 μM RSG-treated group compared to the control group. Significant alteration of several proteins was found, including the up-regulation of calcium-binding, cytoskeletal, and metabolic proteins, concomitant with the down-regulation of antioxidant enzymes. Detailed analyses revealed that RSG induced apoptosis in CCA cells, accompanied by increased caspase 3/7 activities, reactive oxygen species (ROS) generation, and disruption of mitochondrial function. RSG altered the expressions of annexin A1 and antioxidant enzymes, according to Western blot analysis. GW9662, a PPARγ antagonist, did not affect the viability and apoptosis of KKU-100 cells caused by RSG. Immunohistochemistry analysis revealed that PPARγ expression in CCA patients was associated with sex, but not with other common clinicopathological parameters. Its expression did not correlate with patients’ overall survival time. **Conclusions**: RSG induced apoptotic cell death in CCA cells, which was accompanied by increased ROS levels and impaired antioxidant defense. Its apoptosis-inducing effect is independent of PPARγ activation. These findings underscore the therapeutic potential of RSG for CCA treatment.

## 1. Introduction

Cholangiocarcinoma (CCA) is a neoplasm of the biliary epithelium, with a high incidence in Southeast Asia [1]. The predominance in this region is closely associated with chronic infection by *Opisthorchis viverrini*, a liver fluke that induces persistent biliary inflammation, leading to dysregulation of intracellular signaling pathways [2]. The asymptomatic characteristics of early-stage CCA lead to most patients being detected at metastatic or advanced stages, making curative surgery impractical, with chemotherapeutic regimens as the treatment option [3]. Nonetheless, existing chemotherapeutic agents provide limited outcomes due to the emergence of multidrug resistance in the disease [4]. These problems highlight the necessity for novel therapeutic agents for this aggressive cancer.

Rosiglitazone (RSG) acts as a peroxisome proliferator-activated receptor-γ (PPARγ) agonist. Although initially developed for diabetes management, RSG has garnered interest for its anticancer properties, particularly its capacity to modulate the survival of various cancer cells, including those associated with bladder [5], gastric [6], and ovarian malignancies [7]. This information indicates that this medicine has increasing attention in drug repurposing research for cancer therapy. Previous studies have demonstrated that both PPARγ-dependent and -independent mechanisms mediate the antineoplastic property of RSG. PPARγ-dependent activities include inhibition of the PI3K/AKT/mTOR pathway, which resulted from PPARγ increasing transcription of PTEN [8]; suppression of p38 MAPK signaling [9]; inhibition of NF-κB [10]; induction of the apoptosis process by increasing proline oxidase synthesis [11]; and regulation of apoptosis-related molecules. Moreover, RSG could also inhibit cell adhesion molecule 3, which leads to suppression of gastric cancer cell metastasis [6]. Notably, RSG has demonstrated PPARγ-independent anticancer actions in many cancer types. These mechanisms include promoting the mesenchymal–epithelial transition process leading to cell proliferation and metastasis suppression, and promoting tumor necrosis factor-related apoptosis-inducing ligand-induced cancer cell death by reactive oxygen species (ROS)-mediated DR5 up-regulation [12]. These findings emphasize the complex nature of RSG’s anticancer effects.

In the present study, we aimed to examine the anticancer potential of RSG against CCA. The human CCA cell line, KKU-100, obtained from patients with liver fluke-associated CCA demonstrating limited sensitivity to gemcitabine, a standard chemotherapeutic drug for CCA treatment [13], was utilized in the study. This cell line was selected as a typical model due to its derivation from poorly differentiated tubular adenocarcinoma, the common CCA subtype in Thailand [14], hence rendering it a clinically relevant model. The impact of RSG on CCA cell viability was initially investigated. The proteomic profiles of control and RSG-treated cells were subsequently determined to identify the possible targets of the compound. A series of cell-based assessments and Western blot analyses were conducted to validate the target proteins. Additionally, the expression of PPARγ protein in CCA tissues, its correlation with various clinicopathological features, and cumulative survival rates of CCA were also determined.

## 2. Results

### 2.1. RSG Decreased CCA Cell Viability

**Figure 1:**
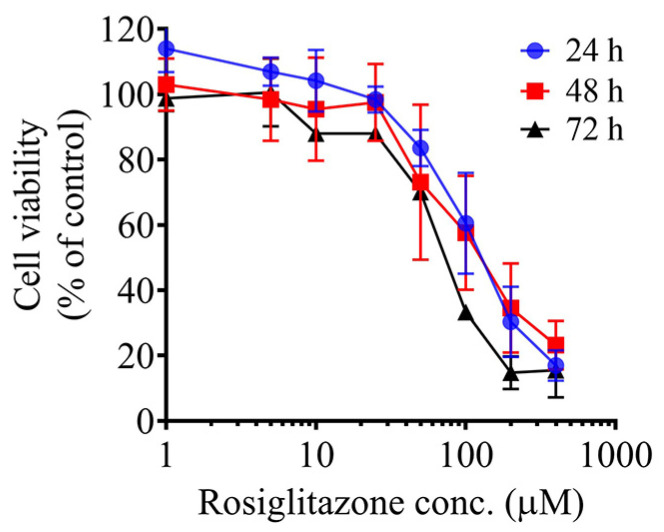
The effect of RSG on CCA cell viability. KKU-100 cells were treated with various concentrations of RSG for 24, 48 or 72 h. The cell viability was determined by MTT assay. Data are the mean ± SD of three independent experiments.

The effect of RSG on KKU-100 CCA cell viability was determined using the MTT assay. The data showed that RSG had a cytotoxic effect on KKU-100 cells in a concentration- and time-dependent manner. The compound exhibited IC_50_ values of 138.9 ± 6.7 μM at 24 h, 115.8 ± 0.5 μM at 48 h, and 66.57 ± 6.7 μM at 72 h, indicating increased potency with prolonged exposure (Figure 1).

### 2.2. Proteomic Profiling Reveals Potential Target Proteins of RSG in CCA Cells

**Figure 2:**
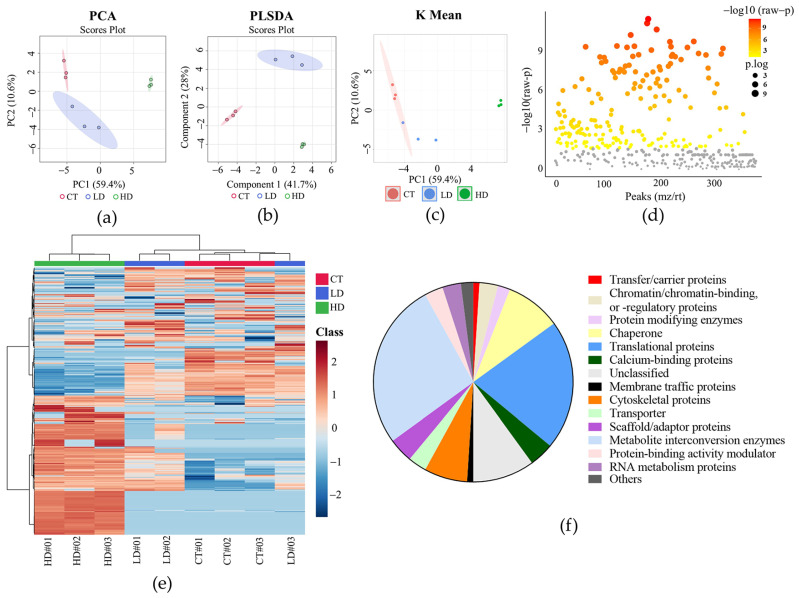
Multivariate, clustering, and functional classification analyses of proteomic profiles in KKU-100 CCA cells. (**a**) Principal component analysis (PCA) plot illustrating the overall variance in protein expression across treatment groups. (**b**) Partial least squares discriminant analysis (PLS-DA) illustrating group differentiation based on proteomic characteristics. (**c**) K-means clustering analysis demonstrating sample categorization based on protein expression similarity. (**d**) Volcano plot illustrating differentially expressed proteins with an FDR-adjusted *p*-value < 0.05, with significantly upregulated and downregulated proteins highlighted. Grey dots represent proteins that are not significantly differentially expressed (raw *p* ≥ 0.1). (**e**) Heatmap depiction of significantly modified proteins among treatment groups, demonstrating expression patterns and sample clustering. (**f**) Pie chart illustrating the functional classification of significantly altered proteins (FDR-adjusted *p*-value < 0.05), grouped by protein class using the PANTHER classification system (version 19.0). CT, control; LD, low dose (25 μM RSG); HD, high dose (100 μM RSG).

To investigate the possible target proteins of RSG in KKU-100 cells, a comparative proteomic analysis was subsequently performed. The cells were treated with 25 µM (low-dose group) and 100 µM (high-dose group) of RSG for 72 h, and the profiles of protein expression of treatment cells were compared with untreated control cells. The results indicated that a total of 513 protein groups were identified across all three experimental groups. Among these, 379 proteins exhibited quantifiable label-free quantification (LFQ) intensities appropriate for statistical analysis, of which 179 protein groups showed statistically significant alterations. Figure 2 illustrates the results of multivariate, clustering, and functional classification analyses of proteomic profiles in KKU-100 cells. Principal component analysis (PCA) and hierarchical clustering heatmap indicated that the proteomic profile of high-dose RSG-treated group displayed a significantly different expression pattern compared to both the control and low-dose groups. The functional classification of the differentially expressed proteins revealed that most of them belonged to groups of enzymes, cytoskeletal proteins, molecular chaperones, histones, and ribosomal proteins. As presented in Table 1, several proteins were markedly upregulated following RSG treatment, particularly in the high-dose group, including cell membrane and structural-related proteins such as actinin, tubulin, profilin, filamin A and B, and vimentin; calcium-binding proteins such as annexin A1, A2, A3, and A5; enzymes such as phosphoglycerate kinase, aldo-keto reductase, protein disulfide-isomerase, aldehyde dehydrogenase, glutathione-S-transferase, NAD(P)H dehydrogenase (quinone) 1, and peptidyl-prolyl cis-trans isomerase A; histones such as HSBC14, H4C1, and H2AJ; chaperones such as HSPA5, HSPA8, HSP90AA1, HSP90B1, and HSP90AB1; as well as other proteins including fatty acid-binding protein 5 and calmodulin. In contrast, numerous proteins exhibited significant downregulation in the RSG-treated groups, including enzymes such as superoxide dismutase, triosephosphate isomerase, aspartate aminotransferase, protein deglycase, lactoylglutathione lyase, thioredoxin, and glutaredoxin-1; lectins such as galectin; and other proteins including neuroblast differentiation-associated protein AHNAK, macrophage migration inhibitory factor (MIF), NEDD8, HSPE1, and S100-A4. These findings suggested that RSG may influence multiple cellular processes in KKU-100 CCA cells, including oxidative stress response, protein folding, cytoskeletal organization, and calcium signaling, via the differential regulation of a wide array of proteins.

**Figure 3:**
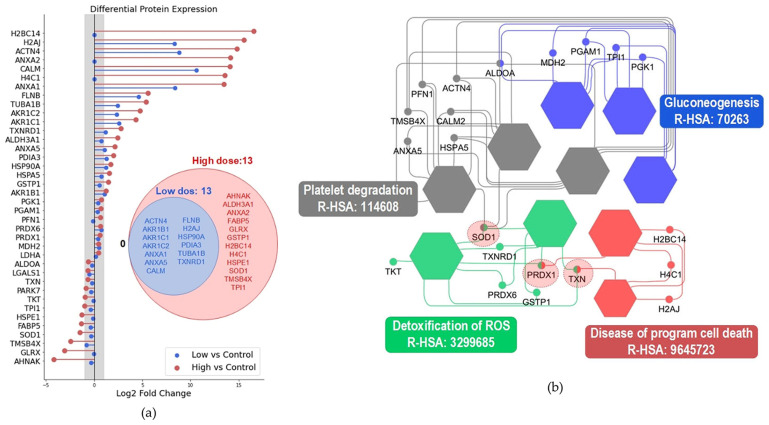
Proteomic comparison and pathway enrichment analysis. (**a**) Differentially expressed proteins in low-dose and high-dose treatment groups compared to control, with shared and distinct proteins (|log2 fold change| > 1) visualized using a Venn diagram. (**b**) Reactome pathway enrichment analysis of significant proteins. Pathways sharing >50% of their constituent proteins are color-coded to indicate functional redundancy, and the top three hub proteins are highlighted with red circles.

To compare dose-dependent proteomic alterations, we examined protein expression changes between the low-dose and high-dose treatment groups relative to the control using log2 fold-change. Proteins with |log2 fold| greater than 1 were considered substantially altered. A Venn diagram was generated to visualize the shared and distinct protein changes between the two treatment conditions (Figure 3a). The analysis revealed that all proteins altered under the low-dose condition were also present in the high-dose group, indicating that the proteomic responses induced at the lower dose were not qualitatively distinct but rather quantitatively amplified under the higher dose.

Hub protein analysis identified superoxide dismutase-1 (SOD1), peroxiredoxin-1 (PRDX1) and thioredoxin (TXN), as the three most central nodes within the protein–protein interaction (PPI) network, suggesting their key regulatory roles in the proteomic landscape. Reactome-based pathway enrichment further revealed four significantly enriched pathways associated with the proteomic dataset (Figure 3b). Among these, two pathways showed the strongest overlap and network connectivity with the hub proteins: (1) Detoxification of ROS and (2) Diseases of programmed cell death. These pathways are closely associated with oxidative stress responses and the regulation of stress-induced cell death. Collectively, these results indicate that redox dysregulation and oxidative stress–mediated cell fate mechanisms likely represent dominant biological processes reflected in the observed proteomic alterations.

### 2.3. RSG Induced Apoptosis and Impaired Antioxidant Defense in CCA Cells

The proteomic analysis revealed significant alterations in proteins that contributed to the regulation of cell death processes. We also observed a significant downregulation of critical antioxidant enzymes implicated in the oxidative stress response. Among the cell death processes, stimulation of apoptosis in neoplastic cells is an important strategy for cancer treatment, and usually the apoptotic cell death is triggered by a cellular oxidative stress condition. Therefore, we further investigated the effects of rosiglitazone on apoptotic pathway activation and oxidative status in KKU-100 cells.

**Figure 4:**
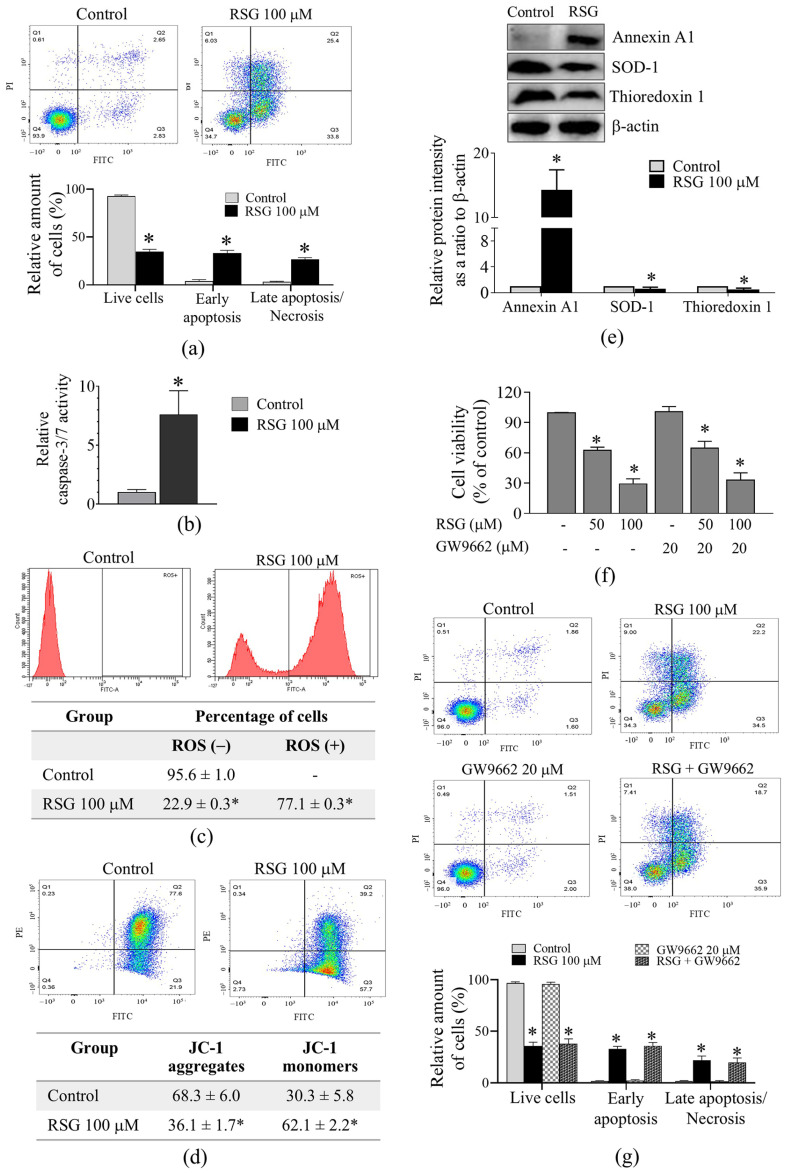
Apoptosis-inducing effect of RSG against KKU-100 CCA cells. (**a**) Quantification of apoptotic cells by annexin V-FITC/PI staining and flow cytometry. The graph shows the percentage of cells in each quadrant: live cells (**lower left**), early apoptotic (**lower right**), and late apoptotic/necrotic (**upper right**). (**b**) Caspase-3/7 activity increased after exposure to 100 μM RSG for 72 h. (**c**) ROS levels assessed by DCFDA fluorescent staining and flow cytometry. (**d**) JC-1 staining revealed increased JC-1 monomer, indicating depolarized mitochondria after 72 h of RSG treatment. (**e**) RSG altered expression of annexin A1 and antioxidant-related proteins. (**f**) Effect of GW9662 on the cytotoxic effect of RSG. (**g**) Effect of GW9662 on the apoptosis-enhancing effect of RSG. Representative images shown are from one of three independent experiments. Data are presented as mean ± SD (*n* = 3–4). *, *p* < 0.05 vs. control. CT, control.

The apoptosis-inducing activity of RSG in CCA cells was evaluated using annexin V-FITC/Propidium iodide (PI) double labeling followed by flow cytometry analysis. The results showed that treatment with 100 µM RSG for 72 h significantly increased apoptotic cell death in KKU-100 cells. The proportion of total apoptotic cells increased from 6.9 ± 1.4% in the untreated control to 59.9 ± 1.7% in the RSG-treated group (Figure 4a). The activity of caspase-3/7 was subsequently assessed, as these executioner caspases are essential markers of apoptosis, and their activation provides direct evidence of the pro-apoptotic action of the compound [15]. The data demonstrated that RSG resulted in a 7.6-fold increase in caspase-3/7 activity in KKU-100 cells (Figure 4b). Given that excessive ROS generation is a dominant initiator of apoptosis [15], and its elevation supports the compound’s role in inducing oxidative stress-mediated cell death, the measurement of ROS level was subsequently conducted. As shown in Figure 4c, RSG-treated KKU-100 cells had an intensive increase in fluorescence intensity when compared with control cells, indicating enhanced ROS production. Considering that elevated intracellular ROS levels can interfere with mitochondrial function, the impact of RSG on the mitochondrial integrity of CCA cells was evaluated using the JC-1 assay. A reduction in the J-aggregate/monomer ratio signifies mitochondrial depolarization. The findings indicated that RSG significantly increased the percentage of cells with depolarized mitochondrial transmembrane potential (Figure 4d). Consistent with these results, Western blot analysis indicated that the expression of annexin A1 was significantly up-regulated, whereas the expressions of antioxidant-related proteins, i.e., superoxide dismutase, and thioredoxin, were significantly down-regulated after RSG exposure (Figure 4e). The data suggested that RSG induced apoptotic cell death and diminished antioxidant protection in CCA cells. To determine whether the cytotoxic and apoptosis-enhancing effect of RSG is due to PPARγ activation, the impact of GW9662, a PPARγ antagonist, on the cell viability and apoptosis of CCA cells was evaluated. The concentration of GW9662 used was 20 μM, which was the concentration previously reported to inhibit PPARγ [16,17], and this concentration had no cytotoxic effect on KKU-100 cells (Figure 4f). The results indicated that GW9662 did not affect the viability and apoptosis of KKU-100 cells caused by RSG (Figure 4f,g), implying that the cytotoxic and apoptosis-enhancing effect of the medicine was facilitated via a PPARγ-independent mechanism.

### 2.4. Expression of PPARγ in CCA Tissues and Its Correlation with Clinicopathological Characteristics and Cumulative Survival Rates of CCA Patients

In the present study, the PPARγ protein expression in CCA tissues and the association of its expression with various clinicopathological features and survival times of CCA patients were also determined. We previously described in detail the clinicopathological and histopathological features of the tumor specimens used in this study [18]. In brief, the study included 111 patients (43 females and 68 males), with a mean age of 63 years. Most of the tumors were categorized as perihilar CCA and were primarily well-differentiated grade G1. A significant percentage of tumors were identified at stage IV. These specimens were utilized to evaluate PPARγ expression by immunohistochemistry in this study. A summary of the clinicopathological characteristics of the patients is presented in Table 2. Detailed data were previously reported in our prior study [18].

**Figure 5:**
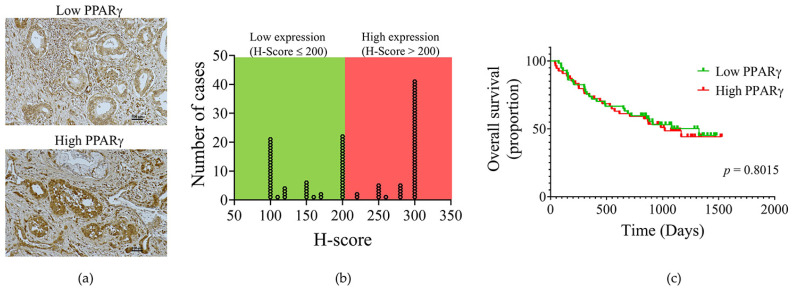
PPARγ expression in CCA and its correlation with cumulative survival rate. (**a**) PPARγ expression in CCA tissues. The scale bar represents 100 μm. (**b**) Distribution of PPARγ scores. (**c**) Kaplan–Meier survival curves stratified by PPARγ expression using the median H-score as the cutoff.

All 111 tissue samples had detectable PPARγ expression, with varied degrees of staining intensity and distribution across cases (Figure 5a). The subcellular location of PPARγ was detected in both the cytoplasm and nucleus. Both cytoplasmic and nuclear staining patterns were assessed for semi-quantitative evaluation. The distribution of PPARγ H-scores is present in Figure 5b. The total protein expression of specimens was classified into two categories using the median H-score (=200) as the cutoff: 55 samples (49.6%) exhibited low expression, whereas 56 cases (50.4%) had high expression. A univariate analysis was performed to determine the association between PPARγ expression in CCA tissues and the established clinicopathological aspects of the patients. The results indicated that no significant relationships were identified between its expression and all assessed characteristics, with the exception of sex (*p* = 0.038) (Table 3). The clinical importance of PPARγ in CCA patients was evaluated by examining the correlation between the expression of this nuclear receptor and the survival time of patients. The Kaplan–Meier analysis demonstrated no association between overall survival and the level of PPARγ expression (Figure 5c).

## 3. Discussion

The development of innovative therapeutic agents for CCA is an important necessity. Among anticancer drug discovery, repurposing current medications as antineoplastic agents has garnered interest because of its significant advantages [8]. In the present study, we demonstrated that RSG induced apoptosis in KKU-100 CCA cells. The proteomic analysis indicated that the drug affected various cellular processes, including proteins associated with the oxidative stress response. Comprehensive molecular analyses revealed that RSG induced apoptotic cell death accompanied by impaired antioxidant defense in CCA cells. Its apoptosis-inducing effect is independent of PPARγ activation. Therefore, this medication may serve as a feasible approach for the treatment of CCA.

Despite cumulative evidence indicating the anticancer efficacy of RSG in several cancer cell lines [5,6,7], its effects on CCA cells have yet to be investigated. This study evaluated the efficacy of RSG in inhibiting the cell viability of CCA cells to confirm the anticancer potential of this medication against CCA. In accordance with previous studies, we found that RSG can diminish CCA cell viability in a dose- and time-dependent manner. The cytotoxic efficacy of the drug in the KKU-100 CCA cell line was comparable to that observed in hepatocellular carcinoma HepG2 cells [19], where treatment with 100 µM RSG for 72 h resulted in a cell viability of about 30% compared to the control group. Mechanistic insights of the anticancer effect of RSG vary among cancer cell types [8]. To identify the possible targets of this medicine in CCA cells, proteomic analysis was employed. Proteomics is an effective tool for examining several biological processes, including cancer biology. The possible cellular targets of the compound of interest can be investigated by performing comparative proteome analysis on treatment and control groups. KKU-100 cells treated with low dose (25 μM) and high dose (100 μM) RSG for 72 h were subjected to proteomic analysis. At the high dose (approximately IC_75_), a substantial proportion of the cells underwent treatment-induced death, making the detected molecular alterations relevant to cell death mechanisms. The results indicated that all proteins altered at the low dose were also affected at the high dose, indicating that RSG engages the same proteomic pathways across concentrations, with higher doses merely intensifying these molecular changes. In this study, we found that RSG significantly altered the expression pattern of several proteins, characterized by the up-regulation of calcium-binding, cytoskeletal, metabolic, histones, and chaperone proteins, concomitant with the down-regulation of antioxidant enzymes and regulatory proteins. These findings suggest the capacity of RSG to perturb calcium signaling, redox balance, protein folding, and cytoskeletal organization in KKU-100 CCA cells.

Annexins are calcium-dependent phospholipid-binding proteins involved in the regulation of diverse cellular processes, including apoptosis, membrane trafficking, and stress and inflammatory responses [20]. This study emphasized the apoptosis-inducing effect of RSG on CCA cells, considering that a crucial characteristic of anticancer agents is their capacity to induce apoptosis in malignant cells, alongside the up-regulation of annexins revealed by proteomic analysis. Among the modified annexin proteins, annexin A1 was prioritized for validation by Western blot analysis due to its documented context-dependent activities in apoptosis [20,21,22]. For annexin A2, although it has been widely characterized as an anti-apoptotic protein, emerging evidence indicates that it can facilitate alternative forms of regulated cell death, particularly autophagy-, ferroptosis-, and pyroptosis-mediated cell death [20]. Therefore, despite its increased expression in our proteomic data, annexin A2 was not selected for further validation in our apoptosis-focused experiments. We recognize the absence of supplementary analyses on annexin A2 and annexin A5 as a limitation of our study. We found that RSG promoted apoptotic cell death of KKU-100 cells. In our model, upregulation of annexin A1 coincided with clear apoptotic phenotypes, suggesting that it may play a pro-apoptotic role under our treatment conditions. In addition to CCA cells, RSG has exhibited apoptosis-inducing action in different cancer cells, including bladder [5] and ovarian cancers [7].

The results obtained from proteomic analysis also demonstrated the pronounced alteration of antioxidant enzymes in RSG-treated cells, potentially signifying a key vulnerability exploited by the medication to trigger the oxidative stress-mediated apoptosis process in CCA cells. We observed that RSG potently induced ROS generation in KKU-100 cells. In accordance with a previous study, RSG has been shown to induce ROS formation in lung cancer cells [11]. Accompanied by increased ROS generation, the mitochondria of KKU-100 cells lose their membrane integrity after exposure to RSG. Loss of mitochondrial outer membrane can trigger the activation of caspases, evidently by increasing the activity of caspase 3 and 7 in RSG-treated KKU-100 cells. Cancer cells typically have elevated basal ROS levels, requiring robust antioxidant defense systems to keep cell survival and proliferation [23]. The antioxidant enzymes are major sources of defensive mechanisms against cellular oxidative stress conditions [23]. Our results demonstrated that RSG not only increased ROS production but also simultaneously attenuated the antioxidant machinery in KKU-100 cells by down-regulation of several key antioxidant enzymes, including superoxide dismutase, and thioredoxin, verified by immunoblotting analysis. Collectively, our findings indicate that RSG induces apoptotic cell death and disrupts antioxidant defense in CCA cells. Future studies incorporating antioxidant rescue or genetic modulation of key redox regulators will be necessary to clarify whether impaired antioxidant defense plays a causal role in RSG-induced apoptosis.

Previous research revealed that both PPARγ-dependent and independent mechanisms facilitated the antineoplastic effects of RSG [8]. Our study further clarified the mechanistic basis of RSG to limit the ability of CCA cells to thrive by interrogating the involvement of PPARγ signaling. Although RSG is recognized as a PPARγ agonist, blockade with the PPARγ antagonist GW9662 failed to abrogate its cytotoxic and pro-apoptotic actions. These findings imply that the cytotoxicity and apoptotic-inducing activity of RSG in CCA cells are predominantly independent of PPARγ. The alternative signaling cascades mediating its effects remain to be further studied. The independence of PPARγ of the apoptosis-inducing effect of RSG in CCA cells is significant, since it may broaden the therapeutic potential of RSG beyond the classical limitation of PPARγ agonists, which have been linked to adverse metabolic and cardiovascular effects [24]. Further investigation to validate the anticancer effect and safety profile of RSG in vivo would be of value.

Although the present study offers important observations, several limitations should be acknowledged. First, the dose of RSG used in this study exceeds clinically achievable levels. This dose was intentionally used to ensure sufficient intracellular exposure for mechanistic interrogation, as is common in in vitro studies of PPARγ agonists [7]. Thus, our findings primarily reflect direct cellular responses rather than clinically relevant drug concentrations. Additionally, the depth of the proteomic coverage is also limitation of our study. Approximately 500 proteins were detected, which mainly represent high-abundance proteins. Regulatory proteins present in low abundance, including transcription factors, kinases, and stress-response mediators, may not have been detected. Therefore, interpretations of drug effects at the pathway level should be made with caution. Future studies employing advanced proteomic approaches (e.g., data-independent acquisition, tandem mass tag, or sample fractionation) will be necessary to attain more comprehensive pathway coverage.

Cumulative data reveals the role of nuclear receptors in cancer development and progression [25]. The expression of some nuclear receptors, including farnesoid X receptor (FXR), correlates with clinicopathologic parameters in CCA patients [18]. Furthermore, the predictive importance of FXR expression also appears relevant in CCA [18]. For PPARγ, its expression is associated with lymph node metastasis and tumor location in colorectal cancer, and the overall survival was marginally elevated but statistically insignificant in cancer patients exhibiting positive PPARγ expression compared to those with negative expression [26,27]. In the present study, a significant correlation was found between PPARγ expression and sex, where females had higher expression, but not other clinicopathological parameters of CCA patients investigated. The expression of PPARγ was not associated with the overall survival time of the patients, suggesting that its expression may not be used as a prognostic predictor of CCA.

In conclusion, the anticancer potential of RSG against CCA was demonstrated. It induced apoptotic cell death and impaired antioxidant defense in CCA cells. These findings underscore the possibility of using RSG for the development of CCA therapy. Additional investigation of supplementary CCA models and further in vivo investigations are necessary to validate the effectiveness of RSG in the treatment of CCA.

## 4. Materials and Methods

### 4.1. Materials

RSG (purity 99.67%), JC-1 dye, and GW9662 (purity 99.79%) were obtained from MedChemExpress Co. (Monmouth Junction, South Brunswick, NJ, USA). Methylthiazolyldiphenyl tetrazolium bromide (MTT) was purchased form Sigma Chemical Co. (St. Louis, MO, USA). Primary antibody against annexin A1 (Cat#DF6254) was obtained from Affinity Biosciences Co. (Cincinnati, OH, USA). Primary antibodies against SOD-1 (Cu/Zn superoxide dismutase, Cat#37385), and thioredoxin 1 (Cat#2429) were obtained from Cell Signaling Technology Co. (Danvers, MA, USA). The secondary antibodies m-IgGκ BP-HRP (Cat#sc-516102) and mouse anti-rabbit IgG (Cat#sc-2357) were purchased from Santa Cruz Biotechnology Inc. (Santa Cruz, CA, USA).

### 4.2. Cell Line and Culture

The CCA cell line, KKU-100 cells, was generously supported by the Cholangiocarcinoma Research Institute, Khon Kaen University. The cells were cultured in Ham’s F12 media containing 10% fetal calf serum, supplemented with sodium bicarbonate, 10 mM HEPES (pH 7.3), gentamicin (100 μg/mL), and penicillin (100 U/mL) in an incubator at 37 °C with 5% CO_2_ and 95% air.

### 4.3. Assay of Cell Viability

The viability of KKU-100 cells after exposure to RSG was determined using the MTT assay, as previously described [28]. The cells were plated in a 96-well plate (7.5 × 10^3^ cells per well), and the culture was allowed to grow overnight at 37 °C. On the next day, the cells were incubated with different doses of test substances for indicated times. For testing the cell viability of combination between a selective PPAR antagonist GW9662 and RSG, the cells were pre-incubated with 20 μM GW9662 for 30 min before being exposed to RSG. Following the complete treatment, the culture was added with MTT solution (0.5 mg/mL) and further incubated at 37 °C for 4 h. Dimethyl sulfoxide was then added, and the optical density was determined at 540 nm using a microplate reader (TECAN, Grödig, Austria).

### 4.4. Protein Extraction and Digestion for Proteomic Analysis

Cell pellets were suspended in 100 µL of 50 mM ammonium bicarbonate buffer and homogenized with metal beads in a Mixer Mill 400 (Retsch GmbH, Haan, Germany) for 3 min over 2 cycles. The resulting lysates were quantified for the protein concentration using the Bradford assay and the protein concentration was adjusted to 7.0 mg/mL. To reduce disulfide bond, protein samples were then treated with 100 mM dithiothreitol at 65 °C for 30 min, after that alkylation was carried out with 500 mM iodoacetamide and keeping the reaction in the dark at room temperature for 20 min. Subsequently, proteins were digested by adding 2.8 µL of 1 mg/mL trypsin and incubated for overnight at 37 °C. The digestion process was stopped by adding 10% formic acid, and the sample was centrifuged at 14,000 rpm for 10 min. The obtained clear supernatant was then transferred to LC-MS vials for further analysis.

### 4.5. LC-MS/MS Analysis

A 20 µL aliquot of each peptide digest was subjected to LC–QTOF analysis on an Agilent 6545XT system equipped with a reversed-phase Agilent Peptide Mapping column (Agilent Technologies, Santa Clara, CA, USA) (2.1 × 150 mm, 2.7 µm), maintained at 60 °C throughout the run. Chromatographic separation of the peptides was carried out using a linear gradient for 85 min at a constant flow rate of 0.4 mL/min. The composition of the mobile phase consisted of solvent A (water containing 0.1% formic acid) and solvent B (acetonitrile containing 0.1% formic acid). Detection was performed using positive electrospray ionization. The ion source was set to a gas temperature of 325 °C, with drying gas delivered at 13 L/min, a nebulizer pressure of 35 psi, a capillary potential of 4000 V, and a nozzle voltage of 500 V. MS data were collected in centroid mode over an *m*/*z* span of 40–1700 for precursor ions and 25–1000 for product ions. Collision energies were automatically adjusted according to the charge state of the precursor ions using the following formulas: CE = 3.1 × (*m*/*z* ÷ 100) + 1 for singly and doubly charged species, and CE = 3.6 × (*m*/*z* ÷ 100) − 4.8 for species with charges of three or higher. Real-time mass calibration was enabled using a reference ion at *m*/*z* 922.0098. Data acquisition was performed using a data-dependent acquisition (DDA) method coupled with label-free quantification (LFQ). MS1 signal intensities were used for protein quantification.

### 4.6. Proteomic Data Processing, Protein Identification, and Statistical Analysis

The raw spectral data files (.d format) obtained from the Agilent 6545XT Q-TOF system were converted to mzXML format using MSConvert, a function within the ProteoWizard toolkit (version 3.0.23299), and OpenMS (version 3.0.0). Protein and peptide identification was subsequently performed using MaxQuant (version 2.6.3) employing the UniProt human protein database, which contained 205,294 entries at the time of analysis. Trypsin was identified as the digestion enzyme, allowing a maximum of two missed cleavage sites. Carbamidomethylation of cysteine residues was defined as a fixed modification, while oxidation of methionine and protein N-terminal acetylation were classified as variable modifications. Label-free quantification (LFQ) was utilized with a minimum ratio count of 1. The false discovery rate (FDR) for both peptide-spectrum matches and proteins identifications was maintained at 0.5%. LFQ intensity matrices produced by MaxQuant were imported into MetaboAnalyst 6.0 for subsequent statistical interpretation. Protein features containing only zero values or a single non-zero entry across the dataset (134 in total) were excluded. To address missing values, zeros were substituted with a value equal to one-fifth of the smallest detected non-zero intensity for the respective protein, thereby reducing distortion in downstream analyses. The proteomic data have been deposited in the JPOST repository (Japan ProteOme STandard Repository) under the dataset identifier JPST004188. The dataset is currently under embargo and can be accessed via https://repository.jpostdb.org/entry/JPST004188 (accessed on 13 November 2025).

### 4.7. Hub Protein Identification and Pathway Enrichment Analysis

Hub protein identification and pathway enrichment analysis were performed as previously described with minor modifications [29]. Briefly, significant proteins obtained from proteomic analysis were imported into Cytoscape (version 3.10.3) for protein–protein interaction (PPI) network construction using the stringApp plug-in (version 2.2.0), applying the Homo sapiens database and an interaction confidence cutoff of 0.4. Hub proteins were then identified using the CytoHubba plug-in (version 0.1), which ranked nodes based on degree centrality. Pathway enrichment analysis was carried out using the ClueGo plug-in (version 2.5.10) with Reactome pathway annotations, applying a Benjamini–Hochberg adjusted *p*-value threshold of 0.05.

### 4.8. Assay of Apoptosis

The apoptosis-enhancing ability of the test substances in KKU-100 cells was evaluated using the Annexin V-FITC/PI Apoptosis Kit (Cat. No. E-CK-A211, Elabscience, Houston, TX, USA) following the manufacturer’s instructions. KKU-100 cells were plated in a 6-well plate (2.5 × 10^5^ cells per well) and exposed to 100 μM RSG, either alone or in combination with GW9662, for 72 h. For the combination treatment, the cells were pre-treated with 20 μM of GW9662 for 30 min before being exposed to RSG. Following the completion of treatment, the cells were collected, rinsed, and stained with annexin V–FITC and propidium iodide dye. The amount of viable and apoptotic cells was quantified using a flow cytometer (BD Biosciences, San Jose, CA, USA).

### 4.9. Assay of Caspase 3/7 Activity

The caspase 3/7 activity was determined using the commercially available Caspase-Glo^®^ 3/7 Assay kit (Promega, Madison, WI, USA) following the manufacturer’s protocols. In summary, KKU-100 CCA cells were cultivated on a 96-well white culture plate (1.5 × 10^4^ cells per well), incubated overnight, and treated with 100 μM RSG for 72 h. Upon complete incubation, the culture cells were added with Caspase-Glo^®^ 3/7 reagent, and further incubated at room temperature for 3 h. The luminescence signal was read with a SpectraMax^®^ L Microplate Reader (Molecular Devices, LLC, San Jose, CA, USA).

### 4.10. Assay of Reactive Oxygen Species (ROS) Formation

The amount of intracellular ROS was assessed utilizing the fluorescent probe, 2,7-dichlorofluorescein diacetate (DCFDA), as previously outlined [30]. KKU-100 cells were inoculated into a 6-well plate (2.5 × 10^5^ cells per well) and incubated overnight. Subsequently, the cells were treated with 100 μM RSG for 72 h and then resuspended in 25 μM DCFDA. Upon completion of treatment, the cells were collected, rinsed, and resuspended in phosphate-buffered saline. The intensity of the fluorescence signal was quantified using flow cytometry.

### 4.11. Assay of Mitochondrial Transmembrane Potential

The function of mitochondria after exposing the CCA cells to RSG was evaluated using the JC-1 dye staining and quantified by flow cytometry as previously described [30]. In brief, KKU-100 cells were cultivated in a 6-well plate (2.5 × 10^5^ cells per well) and incubated overnight. Subsequently, the cells were exposed to 100 μM RSG for 72 h. Thereafter, the cells were stained with JC-1 dye and incubated for 20 min at 37 °C. Subsequent to incubation, the cells were harvested, rinsed, resuspended in phosphate-buffered saline, and analyzed by flow cytometry.

### 4.12. Preparation of Cell Lysate and Western Blot Analysis

KKU-100 cells were cultured into a 6-well plate (Corning, Lowell, MA, USA) (2.5 × 10^5^ cells per well) and permitted to adhere overnight. Subsequently, the cells were incubated with 100 μM RSG for 72 h. Following the completion of treatment, immunoblotting analysis was carried out as previously described [31]. Total cell lysate was obtained using RIPA buffer, and protein concentrations were determined using the Bradford assay. For Western blot analysis, 20 μg of samples was applied to 10% SDS-polyacrylamide gel to separate the proteins and subsequently transferred to a PVDF membrane. Subsequently, the nonspecific binding sites on the membrane were blocked by incubating them with 5% bovine serum albumin. Thereafter, the blot was incubated with specific primary antibodies (1:1000) overnight at 4 °C. The membrane was further incubated with secondary antibodies (1:5000) for 2 h at ambient temperature. The target bands were detected using the Luminata™ Forte Western HRP substrate (Merck Millipore, Watford, UK), and images were captured with the ChemiDoc™ MP Imaging System (Bio-Rad, Hercules, CA, USA).

### 4.13. Human Specimens and Immunohistochemistry Analysis

Immunohistochemistry examination was conducted on the identical 111 paraffin-embedded liver tissue specimens as described in our previous study [18]. The tissue specimens were provided by the biobank of the Cholangiocarcinoma Research Institute, Khon Kaen University, Thailand. The research obtained approval from the Human Research Ethics Committee of Khon Kaen University (HE651204). In summary, the 3 μm tissue sections were deparaffinized, rehydrated, retrieved antigen in citrate buffer (10 mM, pH 6.0), and treated with 3% hydrogen peroxide for suppressing the activity of endogenous peroxidase. To inhibit nonspecific binding, the specimen was treated with 3% bovine serum albumin and further incubated for 2 h. Subsequently, the specimens were incubated with a specific antibody targeting PPARγ at a dilution of 1:200 at 4 °C overnight. Upon complete incubation, the immunosignal was identified using the Dako EnVision HRP-labeled polymer anti-rabbit system (K4003; Dako, Kyoto, Japan), visualized with ImmPACT^®^ DAB substrate (Cat no. SK-4105; Vector Laboratories, Newark, CA, USA), counterstained with hematoxylin, and mounted with VectaMount^®^ medium (H-5000-60; Vector Laboratories, Newark, CA, USA). H-scores, calculated from intensity of staining and the proportion of positive cells, were used for semi-quantitative assessment of PPARγ expression.

### 4.14. Statistical Analysis

Data were analyzed using one-way ANOVA accompanied by post hoc Student–Newman–Keuls or Student’s *t*-test, as appropriate. In the immunohistochemistry study, Pearson’s chi-square test was utilized to assess the correlation between PPARγ expression and the clinicopathological characteristics of CCA patients. The Kaplan–Meier method, along with a log-rank test, was utilising to analyze the disparity in survival durations among groups. A *p* value of less than 0.05 was established as statistically significant.

## Figures and Tables

**Table 1:** Selected significantly altered proteins identified from proteomic analysis of CCA cells based on FDR-adjusted values.

Protein ID	Protein Name	Gene Name	Razor + Unique Peptides	Sequence Coverage [%]	Score	FDR- Adjusted *p*-Value	Relative LFQ Intensity (Fold Change vs. Control)	
							Low Dose Group	High Dose Group
P04075	Fructose-bisphosphate aldolase A	ALDOA ALDA	40	87.6	323.31	<0.01	0.85	0.65
P00338	L-lactate dehydrogenase A chain (LDH-A)	LDHA PIG19	31	72.3	294.43	<0.05	1.12	1.36
P60174	Triosephosphate isomerase (TIM)	TPI1 TPI	22	90.0	258.08	<0.01	0.78	0.50
P29401	Transketolase (TK)	TKT	29	45.6	176.55	<0.01	0.95	0.52
P00558	Phosphoglycerate kinase 1	PGK1 PGKA	32	74.3	162.66	<0.05	1.31	1.69
O43707	Alpha-actinin-4	ACTN4	38	47.0	124.05	<0.01	894.60	57,270.67
Q06830	Peroxiredoxin-1 (EC 1.11.1.24) (Natural killer cell-enhancing factor A) (NKEF-A)	PRDX1 PAGA PAGB TDPX2	14	74.9	118.98	<0.05	1.32	1.53
Q01469	Fatty acid-binding protein 5 (Epidermal-type fatty acid- binding protein)	FABP5	19	94.8	105.17	<0.01	0.77	0.40
P11021	Endoplasmic reticulum chaperone BiP	HSPA5 GRP78	32	46.3	101.18	<0.01	1.65	3.00
P62328	Thymosin beta-4 (T beta-4)	TMSB4X TB4X THYB4 TMSB4	9	81.8	92.62	<0.01	0.58	0.18
P15121	Aldo-keto reductase family 1 member B1	AKR1B1 ALDR1 ALR2	16	59.8	87.42	<0.05	2.11	2.34
Q9BTM1	Histone H2A.J (H2a/j)	H2AJ H2AFJ	8	66.7	84.99	<0.01	638.37	94,593.33
P00441	Superoxide dismutase [Cu-Zn]	SOD1	10	86.4	63.88	<0.01	0.80	0.35
P07737	Profilin-1 (Epididymis tissue protein Li 184a) (Profilin I)	PFN1	9	65.0	54.80	<0.01	0.93	1.55
P40926	Malate dehydrogenase, mitochondrial	MDH2	14	48.8	53.89	<0.01	1.42	1.37
P09382	Galectin-1 (Gal-1) (14 kDa laminin-binding protein) (HLBP14)	LGALS1	10	63.0	50.73	<0.01	0.71	0.64
P18669	Phosphoglycerate mutase 1	PGAM1 PGAMA	10	44.5	50.61	<0.01	1.30	1.61
Q99497	Parkinson disease protein 7 (Maillard deglycase)	PARK7	13	69.3	40.32	<0.01	0.80	0.55
Q04828	Aldo-keto reductase family 1 member C1	AKR1C1 DDH DDH1	16	62.8	39.00	<0.01	6.00	20.14
P10599	Thioredoxin (Trx) (ATL-derived factor) (ADF)	TXN TRDX TRX TRX1	11	76.2	37.28	<0.01	0.85	0.63
P30101	Protein disulfide- isomerase A3	PDIA3 ERP57 ERP60 GRP58	18	34.1	35.80	<0.05	2.40	4.04
P30041	Peroxiredoxin-6	PRDX6 AOP2 KIAA0106	6	34.8	35.36	<0.01	1.66	1.55
P61604	10 kDa heat shock protein, mitochondrial (Hsp10) (10 kDa chaperonin)	HSPE1	15	84.3	34.19	<0.01	0.91	0.41
Q09666	Neuroblast differentiation-associated protein AHNAK (Desmoyokin)	AHNAK PM227	33	18.4	32.16	<0.01	0.80	0.06
P68363	Tubulin alpha-1B chain	TUBA1B	9	25.9	31.61	<0.05	5.43	41.58
P07900	Heat shock protein HSP 90-alpha	HSP90A	16	22.1	31.16	<0.05	2.36	3.26
Q16881	Thioredoxin reductase 1, cytoplasmic (TR)	TXNRD1 GRIM12	7	15.4	30.81	<0.01	2.28	6.79
P08758	Annexin A5 (Anchorin CII) (Annexin V)	ANXA5 ANX5 ENX2 PP4	10	28.1	30.35	<0.01	2.11	4.42
Q9BRL5	Calmodulin	CALM	4	31.3	26.90	<0.01	3076.30	34,463.67
P30838	Aldehyde dehydrogenase, dimeric NADP-preferring	ALDH3A1 ALDH3	14	28.5	25.69	<0.01	1.66	5.48
O75369	Filamin-B (FLN-B)	FLNB FLN1L	23	12.8	23.69	<0.01	24.47	47.48
P09211	Glutathione S- transferase P	GSTP1 FAEES3 GST3	9	50.0	22.69	<0.01	1.48	2.75
P07355	Annexin A2 (Annexin II)	ANXA2	12	42.2	22.04	<0.01	1	36,147.00
P35754	Glutaredoxin-1 (Thioltransferase-1)	GLRX GRX	7	60.4	17.20	<0.01	0.98	0.12
P04083	Annexin A1 (Annexin I)	ANXA1 ANX1 LPC1	14	46.0	14.67	<0.01	657.73	22,478.33
P62805	Histone H4	H4C1	6	46.6	14.62	<0.01	1	24,353.00
P52895	Aldo-keto reductase family 1 member C2	AKR1C2 DDH2	3	57.6	11.38	<0.05	5.08	27.54
Q99879	Histone H2B type 1-M	H2BC14 H2BFE	10	67.5	10.87	<0.01	1	193,283.33

**Table 2:** Summary of clinical and pathological characteristics of the studied patients.

Characteristic	Value ^1^ (*n* = 111)
Gender (Male/Female)	68 (61.3%)/43 (38.7%)
Age (mean ± SD; range)	63 ± 8.2 years (43–83)
Age group	<63 years: 49 (44.1%)
Tumor location	Intrahepatic: 45 (40.5%) Perihilar: 64 (57.7%) Distal: 2 (1.8%)
Tumor grade	G1 (well-differentiated): 93 (83.8%)
Histological subtype	Periductal infiltration (in mass-forming CCA): 44 (39.6%)
Stage	Stage IV: 55 (49.6%)
Mucin production	Present in 91 (81.9%)
Lymph node metastasis (LN)	Positive in 53 (47.7%)
Lymphovascular space invasion (LVSI)	Present in 95 (85.6%)

^1^ Data are presented as number (percentage).

**Table 3:** Relationship between PPARγ expression and clinicopathological parameters of CCA patients.

Parameter		PPARγ ^1^		*p*-Value ^2^
		Low (%) (H-Score ≤ 200)	High (%) (H-Score > 200)	
Sex	Male	39 (57.4)	29 (42.6)	0.038
	Female	16 (37.2)	27 (62.8)	
Age	<63	26 (53.1)	23 (46.9)	0.511
	≥63	29 (46.8)	33 (53.2)	
Tumor location	Intrahepatic	22 (48.9)	23 (51.1)	0.993
	Perihilar	32 (50.0)	32 (50.0)	
	Distal	1 (50.0)	1 (50.0)	
Histological grade	Grade 1	45 (48.4)	48 (51.6)	0.844
	Grade 2	9 (56.3)	7 (43.8)	
	Grade 3	1 (50.0)	1 50.0)	
Histological grade	Well differentiation	45 (48.4)	48 (51.6)	0.578
	Not well differentiation	10 (55.6)	8 (44.4)	
Histological type ^3^	ID	2 (50.0)	2 (50.0)	0.520
	PI	1 (50.0)	1 (50.0)	
	MF	9 (39.1)	14 (60.9)	
	ID + PI	1 (100)	0 (0)	
	ID + MF	16 (66.7)	8 (33.3)	
	PI + MF	20 (45.4)	24 (54.6)	
	ID + PI + MF	6 (46.2)	7 (53.8)	
IPNB	No	30 (43.5)	39 (56.5)	0.101
	Yes	25 (59.5)	17 (40.5)	
Tumor size	<4.5 cm	32 (51.6)	30 (48.4)	0.625
	≥4.5 cm	23 (46.9)	26 (53.1)	
Mucin	Absence	9 (45.0)	11 (55.0)	0.653
	Presence	46 (50.5)	45 (49.5)	
LVSI	Absence	10 (62.5)	6 (37.5)	0.263
	Presence	45 (47.4)	50 (52.6)	
LN	Absence	31 (53.4)	27 (46.6)	0.282
	Presence	24 (45.3)	29 (53.7)	
Tumor stage	Stage I–II	12 (57.1)	9 (42.9)	0.439
	Stage III–IV	43 (47.8)	47 (52.2)	

^1^ Data are presented as number (percentage). ^2^ Significance defined by *p* < 0.05. ^3^ ID: Intraductal; PI: periductal infiltrating; MF: mass forming; IPNB: intraductal papillary neoplasm of the bile duct; LVSI: lymphovascular space invasion; LN: lymph node metastasis.

## Data Availability

The original contributions presented in this study are included in the article/Supplementary Material. Further inquiries can be directed to the corresponding author.

## Supplementary Materials

The following supporting information can be downloaded at: https://www.mdpi.com/article/10.3390/ph19010044/s1, Figure S1: STR profile of KKU-100 cells; Figure S2: Original blot of Figure 4e; Figure S3: Representative microscopic images of KKU-100 cells after exposure to rosiglitazone for 72 h.

## Abbreviations

The following abbreviations are used in this manuscript:

CCA	Cholangiocarcinoma
PPARγ	Peroxisome proliferator-activated receptor-γ
ROS	Reactive oxygen species
RSG	RSG
