# Supplementary material for: Repurposing Rosiglitazone Induces Apoptosis Accompanied by Impaired Antioxidant Defense in Cholangiocarcinoma Cells: Findings from Proteomic and Functional Analyses
Source: Pharmaceuticals (Basel). 2025 Dec 24;19(1):44. doi: 10.3390/ph19010044 (PMC12845233; doi:10.3390/ph19010044)
Supplement: Supplementary file 1 [file pharmaceuticals-19-00044-s001.zip › Supplementary Figure S2--Original blot of Figure 4e.pdf]

Supplementary Figure S2: Original blot of Figure 4e.

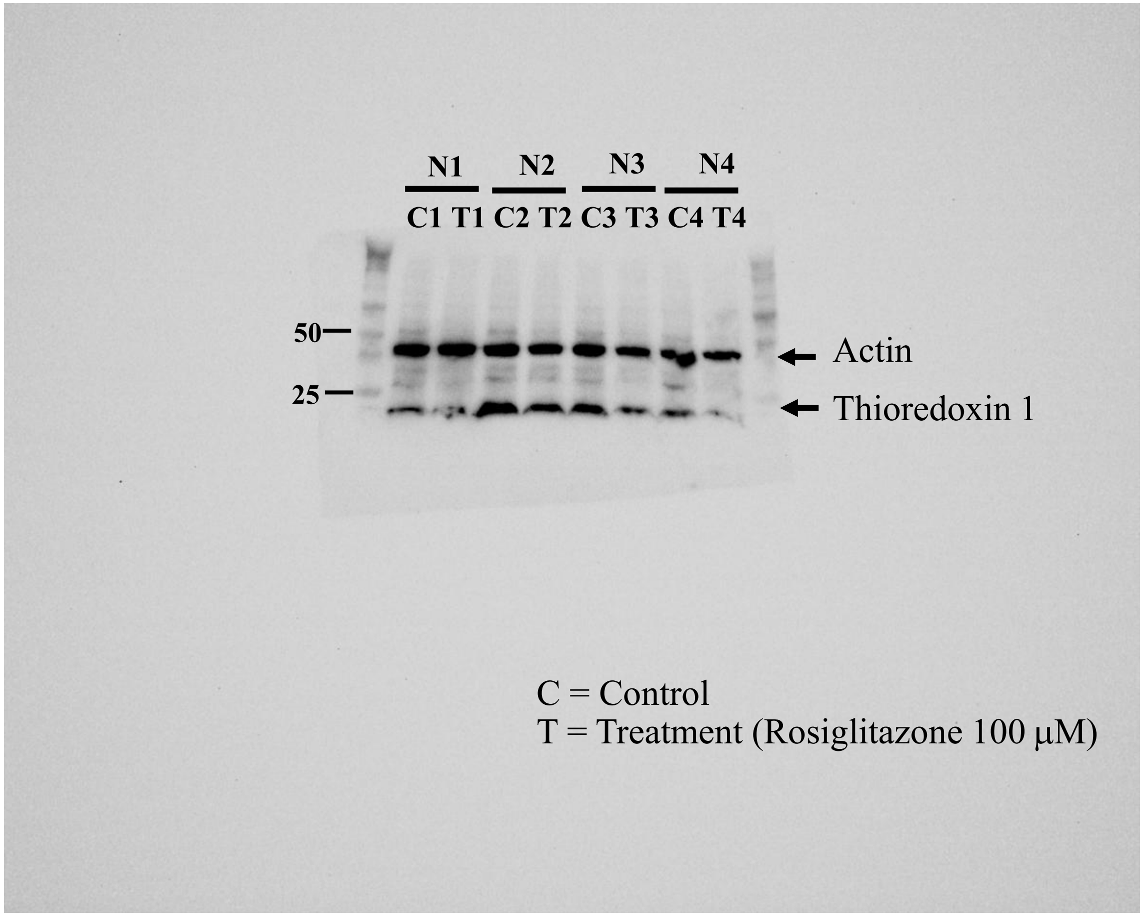

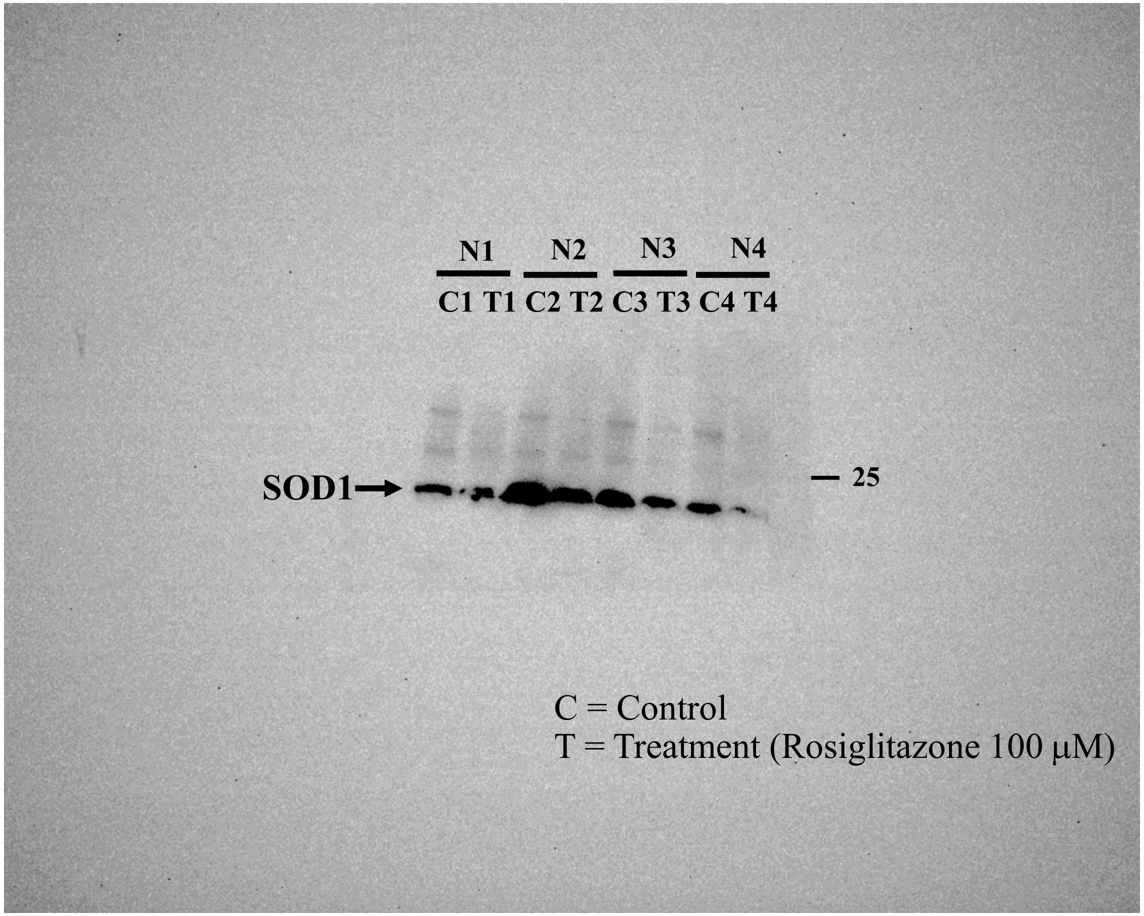
N1
N2
N3
N4
C1 T1 C2 T2 C3 T3 C4 T4
SOD1
25
C = Control
T = Treatment (Rosiglitazone 100 μM)

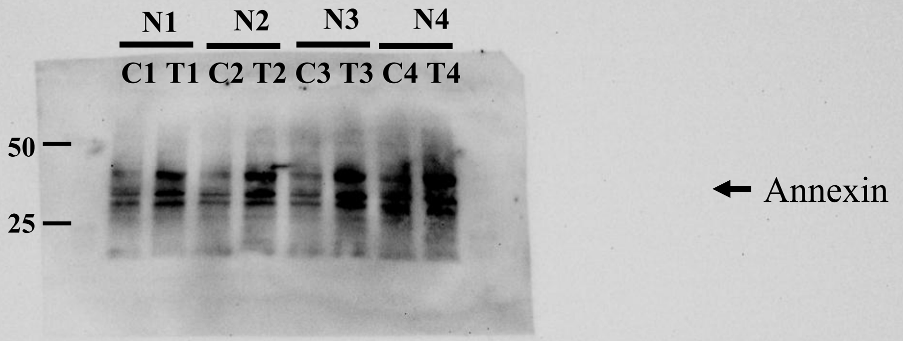

C = Control
T = Treatment (Rosiglitazone 100 μM)
