# Supplementary material for: Repurposing Rosiglitazone Induces Apoptosis Accompanied by Impaired Antioxidant Defense in Cholangiocarcinoma Cells: Findings from Proteomic and Functional Analyses
Source: Pharmaceuticals (Basel). 2025 Dec 24;19(1):44. doi: 10.3390/ph19010044 (PMC12845233; doi:10.3390/ph19010044)
Supplement: Supplementary file 1 [file pharmaceuticals-19-00044-s001.zip › Supplementary Figure S3--Representative microscopic images of KKU-100 cells after exposure to rosiglitazone for 72 h.pdf]

Control

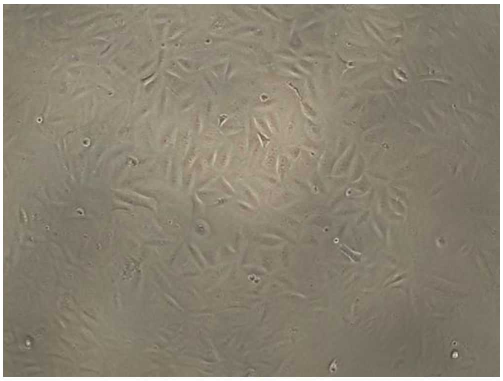

Rosiglitazone 25 μM

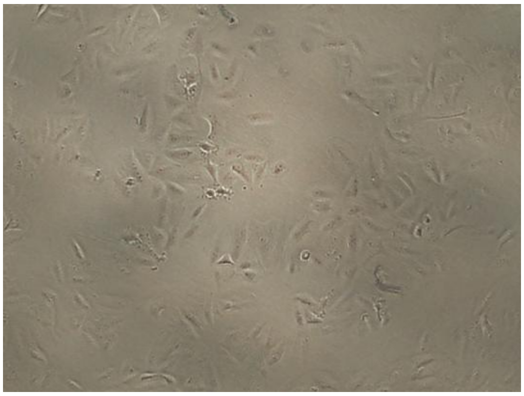

Rosiglitazone 100 μM

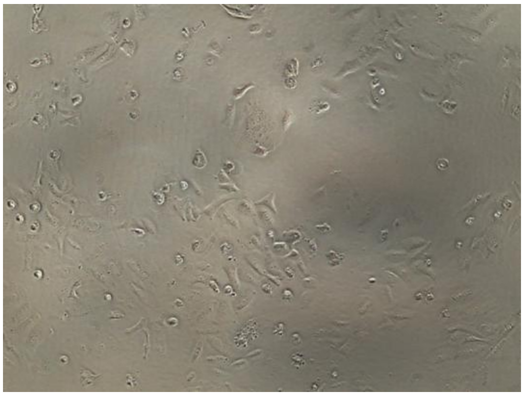

Figure S3. Representative microscopic images of KKU-100 cells after exposure to rosiglitazone for 72 h.
