# Supplementary figures and images for: Repurposing Rosiglitazone Induces Apoptosis Accompanied by Impaired Antioxidant Defense in Cholangiocarcinoma Cells: Findings from Proteomic and Functional Analyses
Source: Pharmaceuticals (Basel). 2025 Dec 24;19(1):44. doi: 10.3390/ph19010044 (PMC12845233; doi:10.3390/ph19010044)

GeneMapper™ ID-X 1.7.2

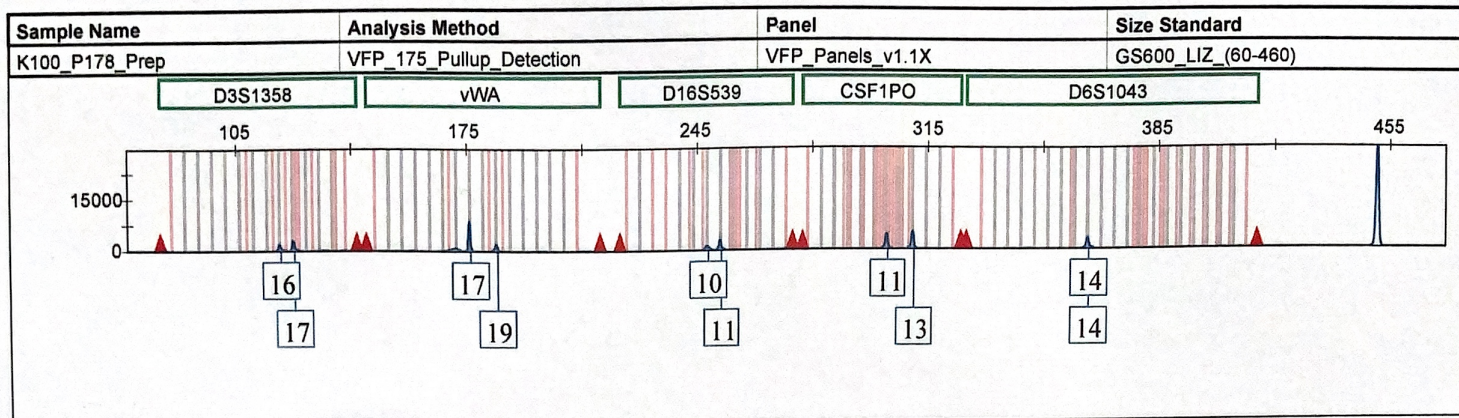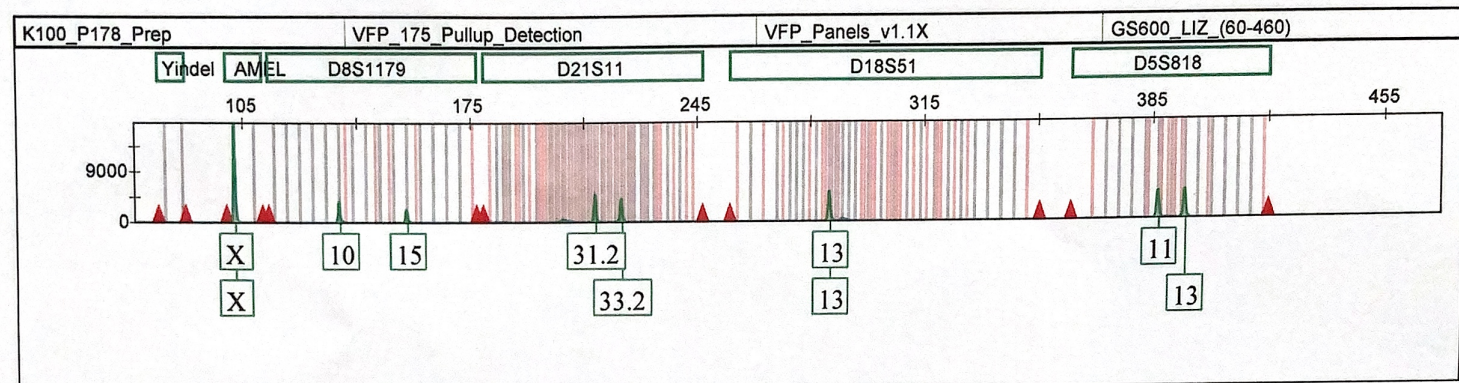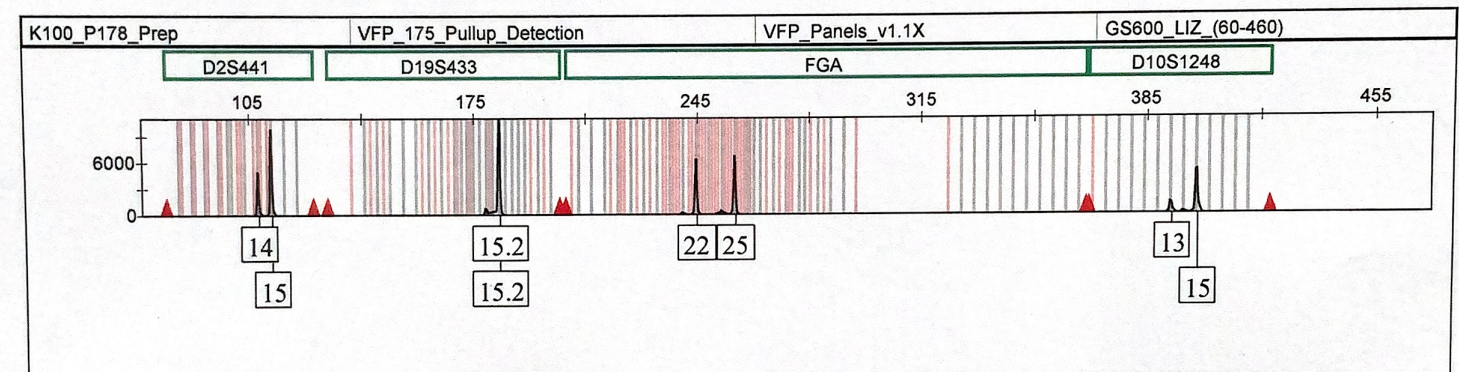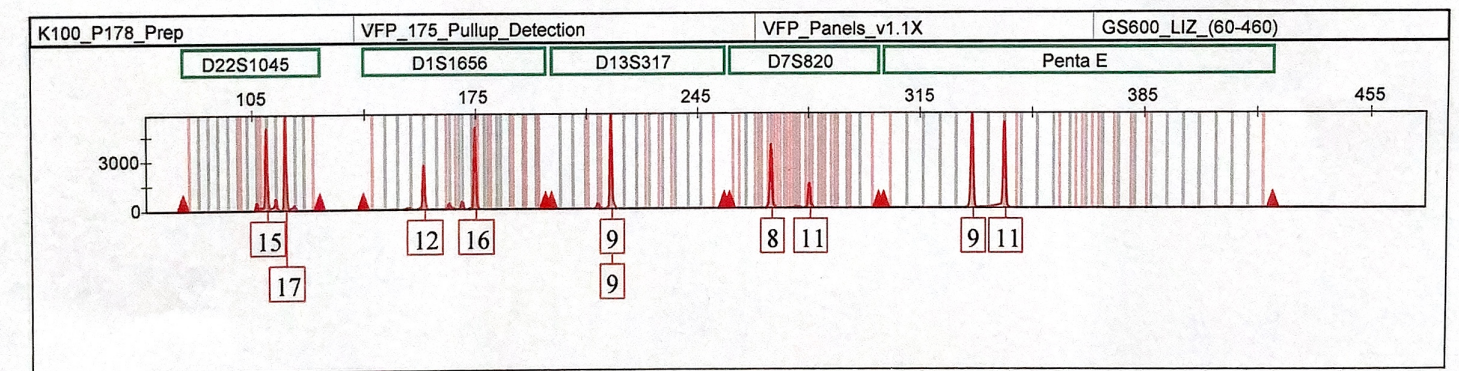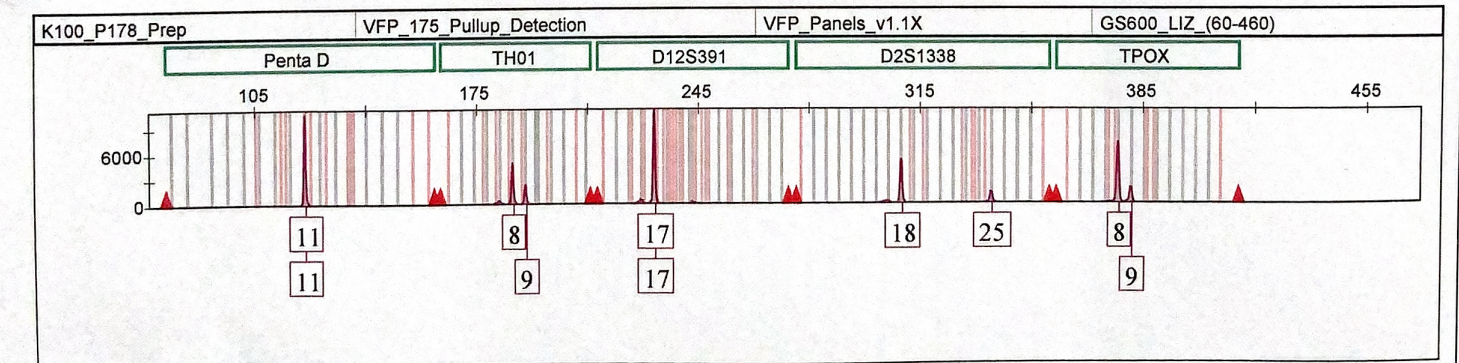

Supplement: Supplementary file 1 [file pharmaceuticals-19-00044-s001.zip › Supplementary Figure S1-STR profile of KKU-100 cells.pdf]
